# Optimized targeted sequencing of cell-free plasma DNA from bladder cancer patients

**DOI:** 10.1038/s41598-018-20282-8

**Published:** 2018-01-30

**Authors:** Emil Christensen, Iver Nordentoft, Søren Vang, Karin Birkenkamp-Demtröder, Jørgen Bjerggaard Jensen, Mads Agerbæk, Jakob Skou Pedersen, Lars Dyrskjøt

**Affiliations:** 1 Department of Molecular Medicine, Aarhus University Hospital, Aarhus, Denmark; 2 Department of Urology, Aarhus University Hospital, Aarhus, Denmark; 3 Institute of Clinical Medicine, Health, Aarhus University, Aarhus, Denmark; 4 Department of Oncology, Aarhus University Hospital, Aarhus, Denmark

## Abstract

Analysis of plasma cell-free DNA (cfDNA) may provide important information in cancer research, though the often small fraction of DNA originating from tumor cells makes the analysis technically challenging. Digital droplet PCR (ddPCR) has been utilized extensively as sufficient technical performance is easily achieved, but analysis is restricted to few mutations. Next generation sequencing (NGS) approaches have been optimized to provide comparable technical performance, especially with the introduction of unique identifiers (UIDs). However, the parameters influencing data quality when utilizing UIDs are not fully understood. In this study, we applied a targeted NGS approach to 65 plasma samples from bladder cancer patients. Laboratory and bioinformatics parameters were found to influence data quality when using UIDs. We successfully sequenced 249 unique DNA fragments on average per genomic position of interest using a 225 kb gene panel. Validation identified 24 of 38 mutations originally identified using ddPCR across several plasma samples. In addition, four mutations detected in associated tumor samples were detected using NGS, but not using ddPCR. CfDNA analysis of consecutively collected plasma samples from a bladder cancer patient indicated earlier detection of recurrence compared to radiographic imaging. The insights presented here may further the technical advancement of NGS mediated cfDNA analysis.

## Introduction

A tremendous progress in the understanding of the molecular processes central to cancer development has occurred during the recent years - a progress fuelled by the development and extensive use of next generation sequencing (NGS). Tumor evolution and differences in cellular composition have been described and multi-region sequencing studies have shown a striking level of intra tumor and intra patient heterogeneity in multiple cancers^1–4^. Furthermore, substantial differences have been observed in the mutational landscapes between primary tumors and distant metastases^5^. These observations question the reliability of the traditional single biopsy procedure for genomic analysis of solid tumors and raise a need for a broad characterization of the tumor burden.

Cell-free DNA (cfDNA) represents DNA originating from dying cells and from active release from viable cells. CfDNA also harbors genetic aberrations from malignant tissue^6,7^ and the short half-life of cfDNA makes it an ideal minimally invasive tool for real-time analysis. The majority of cfDNA, however, originates from dying non-cancerous cells, resulting in very low frequencies of tumor-specific genomic alterations^8,9^. Identification of mutations in cfDNA therefore requires ultra-deep sequencing as a read depth of 1000x would be required to assess a mutation at a frequency of 0.1%.

Circulating tumor DNA (ctDNA) has been detected in cancer patients both prior to and after intended radical surgery and found indicative of later disease recurrence with a positive lead time compared to radiographic imaging^10–14^. Analysis of ctDNA has provided insights into tumor evolution and treatment failure in advanced cancers^9,15,16^. Digital droplet PCR (ddPCR) has been applied extensively to cfDNA as it offers excellent sensitivity and specificity. DdPCR assays are generally designed based on a priori knowledge of tumor-specific mutations and are therefore not suited for detecting new mutations and to study tumor evolution. Furthermore, only few mutations can be assayed as options for multiplexing are limited. NGS has also been used extensively to analyse cfDNA, but is associated with an error rate often superseding the observed frequencies of ctDNA (error rates range roughly from 0.1–1%)^17–19^. An effective method for lowering the error rate of ctDNA sequencing includes incorporation of unique identifiers (UIDs) during library preparation. UIDs are incorporated prior to amplification and reads with identical UIDs thereby originate from the same original DNA fragment. This enables grouping of raw reads by UIDs (UID families) for creation of unique high-confidence reads mimicking original DNA fragments^20–22^. Previous studies have investigated the importance of the size of UID families, but detailed knowledge is lacking on how it is influenced by various parameters^23^.

Bladder cancer (BC) is the 7^th^ most common cancer worldwide with an estimated 430,000 new cases and 165,000 deaths annually^24^. In Danish males and females it is the 4^th^ and 10^th^ most common neoplasm, respectively^25^. Approximately 25% of bladder cancer patients present with muscle-invasive disease of which up to 50% develop metastases^26^. Standard treatment of localized muscle invasive disease is neoadjuvant cisplatin-based combination chemotherapy followed by radical cystectomy, granted patients have an adequate performance status. Metastatic disease is primarily treated with cisplatin-based combination therapy, although immune checkpoint blockade has shown promise as well^27,28^. Patients are monitored by radiographic imaging to assess treatment efficacy and disease relapse.

In this study, we present a custom targeted NGS platform (gene panel) applicable to plasma samples from bladder cancer patients. We characterized the influence of both laboratory and bioinformatics parameters on the efficacy of converting raw reads to unique high-confidence reads.

## Results

### Construction of gene panel for targeted sequencing

**Figure 1:**
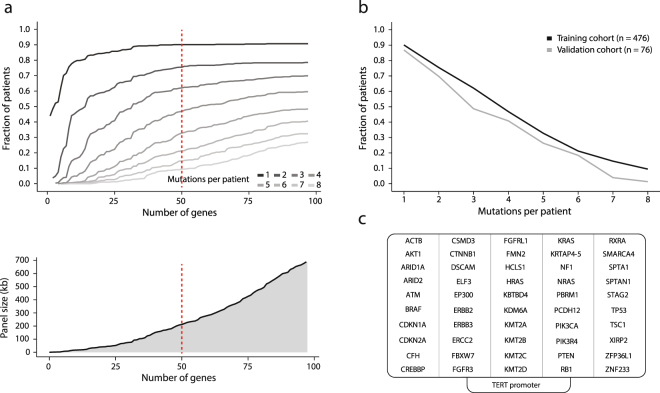
Selection of frequently mutated genes in bladder cancer. (**a**) Upper panel: The fraction of patients with given numbers of mutations detected is displayed for an increasing number of genes frequently mutated in bladder cancer. Genes were ranked according to number of mutations per nucleotide. Lower panel: Size of the gene panel as a function of number of genes included. The red line indicates the number of genes selected for enrichment. (**b**) The fraction of patients with mutations as a function of mutation number per patient is shown for the training cohort used in (**a**) and for a local bladder cancer validation cohort. (**c**) Genes selected for enrichment. The TERT promoter region was added subsequently.

A 51-gene panel for deep targeted sequencing of cfDNA was designed by identifying frequently mutated genes in bladder cancer. Publicly available mutational data of 476 bladder cancer patients from cBioPortal was used, and data was analyzed to assess the probability of detecting mutations while limiting the genomic size of the gene panel (Fig. 1a,b). To enable theoretical detection of three SNVs in more than half the patients, exons of 50 genes were selected for enrichment (approximately 225 kb) (Fig. 1c). Similar results were obtained when applying the same procedure to a local data set (Fig. 1b)^29^. The TERT promoter is frequently mutated in bladder cancer and was therefore included in the final gene panel^30,31^.

### Targeted next generation sequencing using unique identifiers

**Figure 2:**
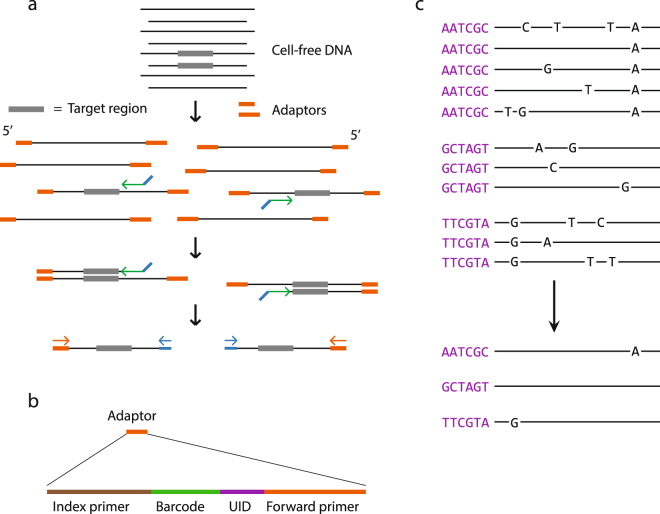
Targeted sequencing process and UID usage. (**a**) Graphical overview of the enrichment process applied in the targeted sequencing approach. Colored arrows represent target-specific enrichment probes and sequencing primers. (**b**) Illustration of sequencing adaptor content. The barcode is used for sample multiplexing. The UID is a stretch of six random nucleotides. (**c**) Reads with identical mapping positions are represented by individual lines and associated UIDs. Reads with identical UIDs are grouped and collapsed to high-confidence reads.

We performed target enrichment of the gene panel by employing a commercial version of anchored multiplex PCR (AMP) for NGS^32^. AMP is based on ligation of universal adaptors to both ends of dsDNA followed by hybridization and elongation of target-specific nested primers with a constant 3′ adapter overhang sequence. DNA fragments thereby contain a universal 5′ adapter sequence and a custom 3′ sequence allowing for large-scale amplification (Fig. 2a,b). The forward adaptors include a UID composed of six random nucleotides (4096 different UIDs). By ligating adaptors to DNA fragments before amplification every DNA fragment will have a unique tag. Upon read mapping, the 5′ mapping positions are inferred along with UIDs to allow for tagging of more unique DNA fragments. Reads with identical mapping positions and UIDs are grouped (UID family) and every nucleotide position analyzed for the most frequently represented nucleotide (Fig. 2c). Collapsing the reads of a UID family into a high-confidence consensus read enables a substantial reduction in sequencing- and PCR errors (Supplementary Fig. 2), while also quantifying the amount of DNA fragments successfully tagged by UIDs before amplification and successfully sequenced. In this optimization project we applied the method to 65 plasma samples obtained from bladder cancer patients.

### Generation of unique reads based on PCR amplification parameters

Identification of low frequency variants requires high target coverage. Achieving high unique coverage, based on high-confidence reads constructed from UID families, requires ultra-high raw coverage and highly accurate grouping and collapsing of UID families (Fig. 2c). Here we sequenced plasma samples to a mean depth of 8124 × (40.7 M reads per sample). UID family grouping and collapsing reduced mean depth to 249 × (3.8 M unique reads per sample). Sequencing details are presented in Supplementary Table 1.

**Figure 3:**
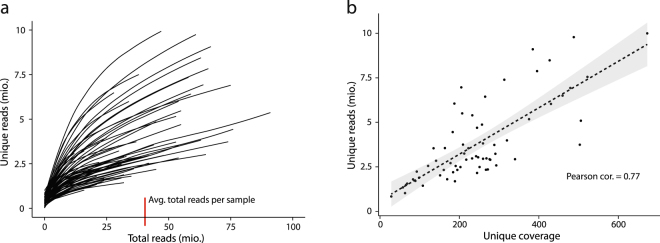
Data distribution for all sequenced plasma samples. (**a**) Total reads obtained and unique reads constructed are displayed for every sample. Lines are constructed from points for every million total reads and correspondingly constructed unique reads. (**b**) Correlation between unique reads and unique coverage. Unique reads were counted without considering UID errors using UMI Tools, thereby corresponding to the unique reads in figure (**a**).

Library preparation was optimized by changing one library protocol parameter at a time to assess the influence on resulting unique reads. Counting the number of unique reads per million raw reads, we observed a profound inter sample difference in terms of effectiveness in constructing unique reads (Fig. 3a). Importantly, we observed that libraries were far from saturated at the current sequencing depth, as the curves representing the relation between unique reads and mapped raw reads did not reach a plateau. This indicated that samples can be sequenced to a greater depth while maintaining the ability to construct additional unique reads, hence increasing the unique coverage (Fig. 3b).

**Figure 4:**
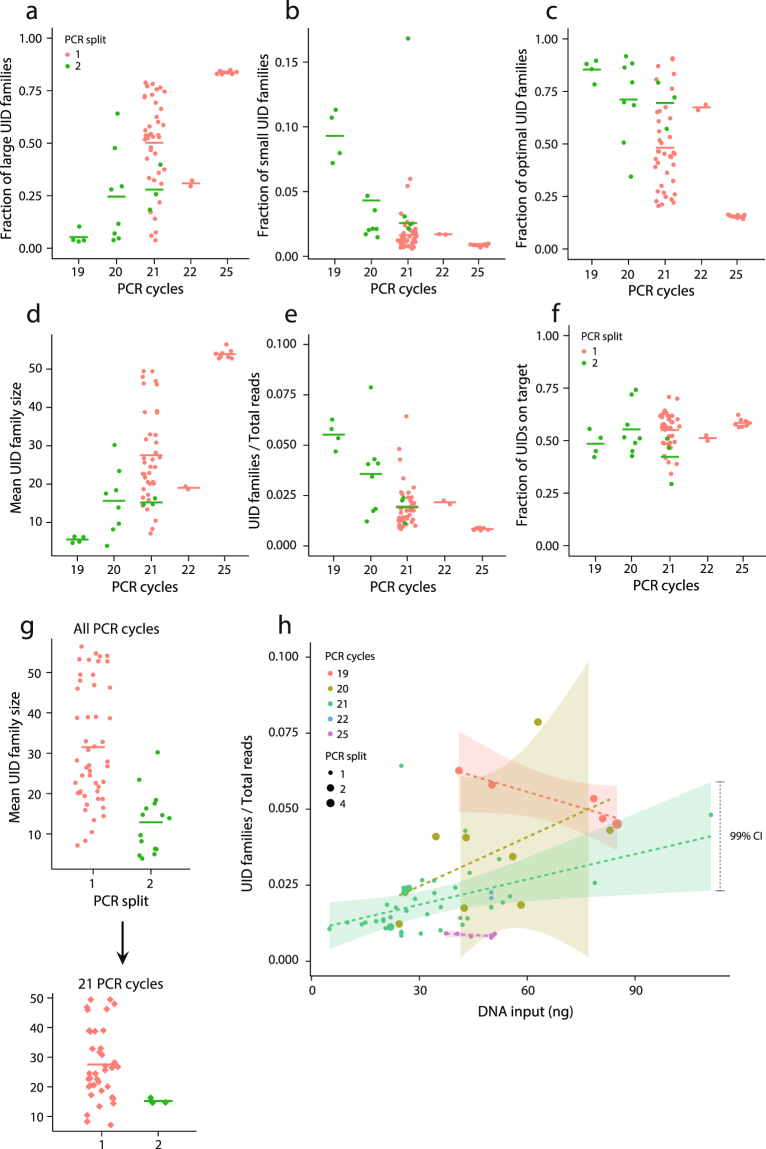
PCR cycles in library amplification and associated UID family composition and efficiency. (**a**) Fraction of large UID families (>50 reads per family) for varying number of PCR cycles. Dots represent single samples; lines represent means per PCR cycle. (**b**) Fraction of small UID families (1–2 reads per family). (**c**) Fraction of optimal UID families (3–50 reads per family). (**d**) The mean size of UID families. (**e**) Total number of constructed and finalized UID families is presented as a fraction of total reads assigned per sample. (**f**) On-target fraction of UID families. (**g**) A number of samples subjected to fewer PCR cycles were split into two PCR amplifications. The upper panel shows the mean UID family size for PCR amplifications for standard and split PCR amplifications. The lower panel shows same comparison for 21 PCR cycles and hence without bias introduced from number of PCR cycles. (**h**) Total number of constructed and finalized UID families is presented as a fraction of total reads assigned per sample relative to DNA input quantified using Qubit. Dashed lines represent linear models and shaded areas represent confidence intervals constructed per PCR cycle number. Dot size indicates PCR amplification split.

The number of cycles used for PCR amplification ranged from 19 to 25. We analyzed the size of UID families for all libraries to determine the impact of varying PCR cycles on library diversity (Fig. 4a,b). As expected, we observed larger fractions of mapped raw reads being lost in overly large UID families when increasing the number of PCR cycles. In concordance, we observed fewer mapped raw reads being lost in small UID families (too few to collapse into high-confidence reads) when performing more PCR cycles. However, the fraction of raw reads qualifying for optimal (family size 3–50) high-confidence read construction was greater for fewer PCR cycles, indicating the non-informative reads from overly large families represent a larger fraction than reads from small families (Fig. 4c). A key component is the average size of UID families, as an optimal size ensures a minimal fraction of raw reads contributes with no new information. We found the average UID family size to be associated with the number of PCR cycles (Fig. 4d) and seemingly contribute to efficient raw to unique read conversion illustrated by a higher fraction of unique reads relative to raw reads (Fig. 4e).

A subset of libraries amplified using 19–21 PCR cycles were randomly selected and run as two parallel amplifications with half input amounts and subsequently combined to one library. Comparing libraries amplified in one or two tubes indicated that splitting the amplification increased complexity (Fig. 4g). This indication persisted when comparing only libraries amplified using 21 PCR cycles. To further assess the influence of amplification we compared the on-target rate of reads, but found no significant difference (Fig. 4f; p = 0.12). To determine the impact of the DNA input amount, we compared the fraction of unique reads relative to mapped raw reads for varying input amounts - stratified for PCR cycles (Fig. 4h). For inputs ranging from 5–111 ng we observed no consistent increase in the efficiency of raw to unique read conversion when stratifying for PCR conditions. The efficiency of read conversion however differed based on PCR conditions.

### Validation of targeted NGS by digital droplet PCR

**Figure 5:**
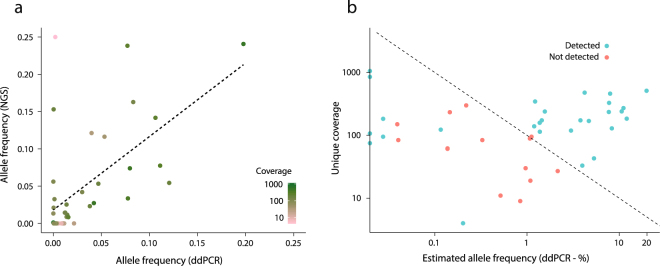
Comparison of targeted NGS and ddPCR. (**a**) A subset of plasma samples subjected to targeted NGS was analyzed previously and mutations were detected using ddPCR assays. (**a**) Allele frequencies obtained using NGS compared to allele frequencies estimated using ddPCR for identical genomic positions (mutations). The dashed line represents a linear model. The color code for NGS coverage is shown to the right. (**b**) Obtained unique coverage (NGS) and estimated allele frequencies (ddPCR) are presented for identical genomic positions (mutations). DdPCR is used as gold standard and detection status refers to NGS data. The dashed line represents the unique coverage theoretically necessary to discover a mutation using NGS (based on the estimated allele frequency using ddPCR).

A subset of plasma samples was previously analyzed for mutations using digital droplet PCR (ddPCR) assays^33^. Fourteen genomic positions were identified for cross-platform analysis by comparing genomic positions targeted by ddPCR assays and genomic regions enriched in the NGS approach. Multiple plasma samples were analyzed using targeted NGS for some of the patients harboring shared positions. These positions thereby contributed to the analysis multiple times. In total, 68 instances of shared positions were analyzed −42 were detected by at least one platform. Initially, we compared the detected allele frequencies in NGS and the estimated allele frequencies in ddPCR (Fig. 5a; Pearson’s cor. = 0.60). Using ddPCR as gold standard, we evaluated the sensitivity of the targeted NGS approach based on the number of ddPCR detected mutations observed in the NGS data. We detected 38 mutations using ddPCR and 24 of these were detected using NGS (63.2%). The theoretical coverage needed for detection of a given variant is shown in Fig. 5b. Mutations detected using ddPCR were mostly detected by NGS when theoretically sufficient coverage was obtained. However, some mutations were detected despite the coverage being lower than the theoretically needed level. This may be due to random subsampling, as withdrawing e.g. 100 reads from a population containing 1000 reads; hereof one mutated, may or may not include the mutated read. Remarkably, four mutations not detected using ddPCR were detected using NGS. Two were detected in other plasma samples from the same patients using ddPCR. The other two were detected at low allele frequencies (0.095%; 0.118%), but validated by identification in associated primary tumor samples.

### Influence of sequencing- and PCR errors in UIDs on mutation calling

Recent targeted NGS studies utilizing UIDs have primarily considered UIDs to be error-free and thereby evaluated every unique UID as truly unique. Sequencing- and PCR errors however also occur in the UID sequence^34^. To assess the influence of UID errors, we identified mutations in all plasma samples using a single sample mutation caller. Standard grouping of UID families enabled detection of an unexpected amount of mutations based on few alternate allele counts (Supplementary Fig. 1). Implementing UMI Tools in our data processing pipeline and thereby accounting for UID errors greatly reduced the number of variants based on low alternate allele counts^34^. To assess eventual loss of true mutations, we checked how many of the ddPCR mutations were detectable using the two different methods for clustering UID families – the same mutations were detected, indicating no loss of true mutations (Supplementary Fig. 1).

### Targeted NGS of plasma DNA compared to clinical data

**Figure 6:**
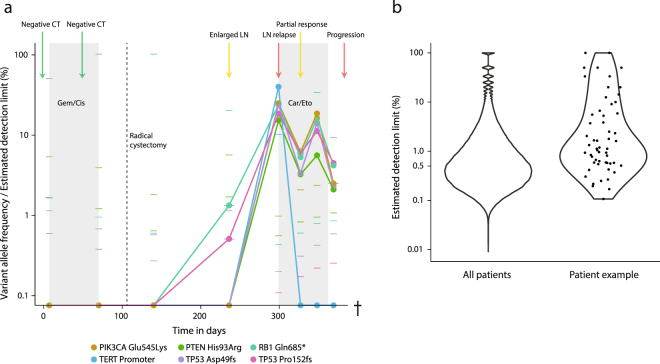
Disease course monitoring using cfDNA analysis for a patient with muscle invasive bladder cancer. (**a**) Eight plasma samples were subjected to NGS during the disease course of the patient. Dots and lines represent observed variant allele frequencies for all identified mutations. Narrow horizontal lines represent the estimated detection limit at the given genomic position in the given sample. Clinical events based on radiographic imaging are marked at the top. Shaded areas represent chemotherapy treatments. (**b**) Distribution of the estimated detection limit for all enriched genomic positions in all patients and the specific genomic positions shown in (**a**).

We analyzed sequencing data in relation to clinical data of a patient with muscle invasive bladder cancer. The patient was treated with neoadjuvant chemotherapy followed by radical cystectomy and diagnosed with lymph node metastasis approximately six months after surgery. We identified six mutations using our 51 gene panel in the primary tumor at diagnosis. We tracked these mutations in eight plasma samples and called new mutations by comparing every plasma sample and germline of the patient. However, no new mutations were identified. Two mutations identified in the tumor were detected in the plasma at approximately 1% frequency four months after radical cystectomy. Concurrent radiographic imaging displayed an enlarged lymph node with ambiguous interpretation, however not sufficient for initiation of treatment. The frequency increased considerably until radiographic and tissue biopsy diagnosis of a lymph node metastasis two months later. Additional mutations originally identified in the tumor were also identified at the time of metastasis. High levels of ctDNA were detectable throughout the remainder of the disease course (Fig. 6a). The estimated detection limit was highly variable between genomic positions considered for the patient - for the majority of positions we were capable of detecting ctDNA at a fraction of 0.5–1%. The detection limits were significantly higher than the distribution of detection limits in all positions in all samples in the study (p < 0.001) (Fig. 6b).

## Discussion

CtDNA analysis has emerged as a promising tool in cancer research. Detection of rare mutations has been associated with treatment response and early detection of disease relapse in multiple cancer types. The rarity of the mutations however necessitates extraordinarily deep sequencing and gene panels targeting only few genes are preferable to avoid high costs, which may limit the eventual clinical applicability. In this study, we enriched for exons of 50 genes frequently mutated in bladder cancer, thereby expecting to detect three mutations in more than 50% of bladder cancer patients (Fig. 1c). Opting for better assay sensitivity, but similar sequencing costs would provide less data per region and hence an inferior limit of detection. Previous ctDNA sequencing studies have primarily used capture-based approaches. Detection limits have been reported at 0.1% or below^35,36^. Data allocation resulting in read-depths of approx. 10,000 have been reported, however the combined size of regions to be enriched range from a few genes to 300 kb^37,38^. A number of studies have used amplicon-based approaches^7,39,40^. Although comparable results have been obtained, the nature of amplicon based enrichment produces more PCR duplicates, especially when obtaining high coverage. Implementation of UIDs may however resolve the true complexity present in a sample. Methodological comparisons between capture- and amplicon based enrichment indicate coverage uniformity may be better for capture-based enrichment, while on-target rate may be better for amplicon-based enrichment^41^. AMP generates one random end for DNA fragments to be sequenced, but in this study it displays inferior coverage uniformity levels compared to capture-based enrichment^32^. Previous studies have also used whole genome sequencing to assess copy number alterations (CNAs) in cfDNA^42,43^. However, identifying CNAs in plasma involves a comparison between read distributions instead of the simpler present/not present situation of mutational analysis. Overly large amounts of sequencing data are therefore needed unless the ctDNA fraction is high^44^.

In order to achieve an optimal detection limit for ctDNA analysis, a reduction in the standard NGS error rate is necessary. We accomplished this through implementation of UIDs and conversion of raw reads in UID families into unique reads. We found that fewer PCR cycles and splitting of PCR amplifications improved the conversion of raw reads into unique reads. It may be feasible to reduce PCR cycles even further for improved conversion of raw reads to unique reads. However, reducing amplification would disqualify a large proportion of UID families, as they would not satisfy the criteria of a family size of three reads. DNA input amount did not affect the data quality to a large extent, demonstrating that the used inputs are sufficient to obtain the reported unique read depths. However, rare mutation detection may improve with larger input amounts, as a DNA fragment containing the rare mutation is more likely to be present.

Recently, UID errors have been reported and tools to circumvent these have been developed^34^. Utilizing UMI Tools to take UID errors into account, we observed a substantial reduction in low frequency mutations presumed to be false positives based on ddPCR measurements.

The average estimated detection limit was 0.4% when allocating approximately 40 million reads per sample. A recent study by Vandekerkhove *et al*. showed promising results for ctDNA analysis for patients with advanced bladder cancer despite inferior detection limits^45^. For the majority of samples the UID family sizes were optimal^23^ and additional sequencing data would allow for efficient construction of more unique reads, i.e. allocation of 100 million reads per sample would provide an even higher sensitivity (0.18% estimated). It would however imply a higher cost per sample. The amount of sequencing data and hence cost of analysis per sample thereby dictates the detection limit for the sample. Comparisons with ddPCR further augmented this observation. The majority of mutations detected using ddPCR were also detected using the NGS approach. However, when mutations were not detected using NGS, the theoretical detection limit was insufficient. For a subset of the comparisons with extraordinarily high estimated detection limits, we were able to detect mutations using NGS not detected using ddPCR. These mutations were all present in the associated tumor samples. Overall, our NGS approach has inferior sensitivity at the current data allocation compared to ddPCR. Detection of rare mutations across platforms may however be biased by subsampling making minor differences difficult to interpret. It is possible to design ddPCR assays for multiple a priori known mutations, however eventually the cost becomes an issue. Furthermore, multiplexing ddPCR is not trivial and larger amounts of cfDNA may be needed to assay multiple mutations (compared to applying an NGS approach) making ddPCR mainly applicable to few mutations. Our NGS approach offers a more comprehensive mutational analysis, however with a lower sensitivity unless a large amount of sequencing data is allocated per sample. The patient example displays how mutations may be present at different frequencies in the circulation at different time points. The sensitivity for ctDNA detection may therefore be greater using NGS due to the concurrent analysis of multiple mutations. Furthermore, recent studies have highlighted the presence of heterogeneous mutations in the circulation^46^, which would be missed using ddPCR as mutations present in the tumour at a high frequency primarily are targeted due to being more frequently detected in the circulation^39^. The patient example further shows some of the clinical utility that may be derived from plasma NGS. Four months after radical cystectomy, two mutations originally present in the tumor were identified at a low frequency. Simultaneous radiographic imaging showed an enlarged lymph node with an ambiguous interpretation. Extraordinary follow-up imaging was therefore performed two months later revealing a large lymph node metastasis further confirmed by a tissue biopsy. Utilizing NGS data may have confirmed the ambiguous lymph node as a metastasis, and may thereby have led to initiation of treatment two months earlier. This is in line with findings from other studies indicating plasma DNA based NGS may be a valuable tool to monitor cancer patients^10,37,47^. It may additionally shed light on disease pathogenesis and in the future guide therapy selection in patients with a high ctDNA burden^45^. Further studies are however needed to evaluate the significance of discovering metastatic relapses and assessing treatment efficacy at an earlier time point.

## Materials and Methods

### Patient samples and processing

Plasma samples were obtained from patients diagnosed with muscle invasive bladder cancer receiving neoadjuvant chemotherapy prior to radical cystectomy and from patients diagnosed with metastatic bladder cancer receiving chemotherapy. Thirty-eight patients were enrolled between 2013 and 2017 at Aarhus University Hospital, Skejby, Denmark and treated according to the national guidelines with 4 series of gemcitabine/cisplatin neoadjuvant chemotherapy before radical cystectomy and 6 series of second-line chemotherapy in case of metastatic disease. All patients provided written informed consent and the study was approved by The National Committee on Health Research Ethics (#1302183).

Forty mL EDTA blood was collected at each visit and before each cycle of chemotherapy and processed within 1.5 hours. Samples were centrifuged at 3000× g for 10 min and stored at −80 °C. Upon usage, plasma samples were thawed at room temperature. Samples were processed for cell-free DNA extraction using the QIAsymphony Circulating NA kit (Qiagen), however with addition of 10% ATL lysis buffer followed by one hour incubation and a five minute centrifugation upon thawing (previously described in^33^). DNA was eluted in 60 µL ddH_2_O in DNA Low Bind Tubes (Sarstedt).

Germline DNA was extracted from buffy coat leucocytes taken upon inclusion/diagnosis. Tumor samples were obtained from TUR-B before initiation of neoadjuvant chemotherapy and DNA was extracted as previously described^33^.

### Sequencing panel design

Mutational data from 476 bladder cancer samples was queried from cBioPortal^48^. Mutational events were summarized for every gene and the number of events per nucleotide was calculated and used for ranking genes (only exons were considered). Calculations and associations based on increasing number of genes were based on this ranking.

The fraction of patients with detectable mutations using a 51-gene panel was validated using mutational data from a previous study using the same procedure as stated above^29^.

### Library preparation and sequencing

Libraries were prepared from 5–111 ng DNA (Qubit) according to the manufacturer’s instructions (Nugen Target Enrichment). Samples were initially concentrated using a DNA120 Speedvac system (ThermoSavant) to the allowed input volume of 10 µL. End repair and an optional repair step for particularly damaged DNA was then performed followed by adaptor ligation. A bead purification step was then performed using a SPRI ratio of 1.0 (0.9 for samples with 25 PCR cycle amplification) to allow for inclusion of small fragments. Samples were subjected to target enrichment followed by two sequential bead purifications. Lastly, libraries were amplified using 19–25 PCR cycles and subjected to two sequential bead purifications using a SPRI ratio of 0.9 to ensure removal of sufficient adaptor dimers. A subset of samples were randomly selected and split prior to amplification. Samples were combined again after amplification. Libraries were pooled eight at a time and single-end sequenced (150 bp) on a Illumina NextSeq 500 (High output) with 15 base index reads to ensure reading of the UID sequence.

### Data processing and UID handling

Fastq files were generated using Illumina’s bcl2fastq v2.18.0.12 using the “–use-bases-mask Y*,I8Y6N*” option to split libraries using the eight base index and keeping the six base UID as an additional read. The UID sequence was added to the read name for each sequence in the fastq file. Individual reads were mapped to hg19 using BWA mem (v0.7.5a) keeping only primary mappings. UMI-tools (v0.3.6) in group mode and directional method were used to cluster the identical UID-genomic-position tags including those with minor sequence variations attributable to PCR errors (thereby originating from the same molecule in the library)^34^. These clusters were numbered and added to the read name. A consensus sequence for each UID cluster was calculated by the umi-consolidate software (https://github.com/aryeelab/umi) and mapped to the hg19 genome. Only clusters of three or more reads were kept in the final alignment to increase the validity of each base-call in the consensus sequence.

### Tumor and germline sequencing for a subset of patients

#### Whole exome sequencing

Approximately 100 ng was used for libraries of tumor and germline samples. Libraries were captured using SeqCapEZ MedExomeV1_hg19 or MedExomePlusV1_hg19 (Roche). FastQ files were processed according to the GATK Best Practices as previously described^33^.

### Digital droplet PCR

Seven patients were included in a recent study where mutations were quantified using ddPCR^33^. Assays were validated using associated tumor and germline samples as positive and negative controls. Zero positive droplets were accepted in germline samples, hence justifying the interpretation of one single positive droplet as a positive signal for mutation assays. Quantification of total DNA was performed for all plasma samples using regions at chromosome 16 and 3, which are rarely subject to copy number variations. Allele frequencies were estimated from mutated DNA relative to total DNA.

### Variant calling

Variant calling for stand-alone plasma samples was performed using MuTect2 with artifact detection mode enabled. Strand filter was enabled to reduce false positive variants originating from only one strand. Default settings were utilized for the remaining parameters. Variants called in more than three samples were discarded to minimize systematic errors. Associated tumor and germline exome sequencing data was utilized for the patient example and somatic variants were called using MuTect2 with default settings.

All variants were annotated using SnpEff and validated using the Integrative Genomics Viewer^49,50^. Bam-readcount was used to count the number of A/C/G/T’s in plasma samples at positions with identified somatic alterations from tumor and germline samples. Estimated detection limits for positions of interest were defined as one divided by the unique coverage.

### Estimation of detection limit with additional sequencing data

The increase in detection limit was estimated based on results of plasma samples amplified using 19/20 PCR cycles. Unique reads obtained for increasing numbers of total reads were extracted as presented in Fig. 3a. A model was made based on the average unique reads per total reads (unique reads ~ squareroot(total reads). The sample-specific values, the average values for all samples and predicted values based on the model are plotted in Supplementary Fig. 3. Using the model, we predicted 6,572,546 unique reads when allocating 40 million reads (the average for all samples) and 10,555,434 unique reads when allocating 100 million reads. For Fig. 3b we created a linear model based on the association between unique reads and unique coverage. Utilizing this model the samples amplified using 19/20 PCR cycles (6,572,546 unique reads) resulted in a mean unique coverage of 372×, while the same samples with additional sequencing data (10,555,434 unique reads) resulted in 551×.

### Calculation of error rates

All gene panel positions were counted for representations of A/C/G/T’s using Bam-readcount. Positions in the gene panel also present in dbSNP were discarded. Positions with read depths <20 and <200 for unique and raw reads respectively were not considered to avoid inflated error rates due to low coverage. To circumvent bias introduced by systematic errors in library preparation or data processing, positions with error rates >10% were discarded. Error rates were calculated as the fraction of non-reference bases relative to total bases. Sample-wise error rates were calculated as the mean error rate of all considered gene panel positions for every sample.

### Statistics

On-target rates were compared using the non-parametric Kruskal Wallis test. Estimated detection limits for the presented patient example were compared to the estimated detection limits for all samples using the Wilcoxon rank sum test.

### Data availability statement

All data is available from the corresponding author upon request.

## Electronic supplementary material

Supplementary figures and table
