# Supplementary material for: Optimized targeted sequencing of cell-free plasma DNA from bladder cancer patients
Source: Sci Rep. 2018 Jan 30;8:1917. doi: 10.1038/s41598-018-20282-8 (PMC5789978; doi:10.1038/s41598-018-20282-8)
Supplement: Supplementary file 1 — Supplementary figures and table [file 41598_2018_20282_MOESM1_ESM.pdf]

Optimized targeted sequencing of cell-free plasma DNA from bladder cancer patients

Emil Christensen[1‡], Iver Nordentoft[1‡], Søren Vang[1], Karin Birkenkamp-Demtröder[1], Jørgen Bjerggaard Jensen[2,3], Mads Agerbæk[4], Jakob Skou Pedersen[1], Lars Dyrskjøt[1]*.

1 Department of Molecular Medicine, Aarhus University Hospital, Denmark

2 Department of Urology, Aarhus University Hospital, Denmark

3 Institute of Clinical Medicine, Health, Aarhus University, Denmark

4 Department of Oncology, Aarhus University Hospital, Denmark

‡ Authors contributed equally.

* Corresponding author: Lars Dyrskjøt, PhD, Department of Molecular Medicine, Aarhus University Hospital, Denmark. Email: lars@clin.au.dk.

Supplementary figures and table

Supplementary figure legends

Supplementary Fig. 1 - Optimized UID family clustering. The number of mutations with low alternate allele counts is displayed for all plasma samples. Standard: Clustering of reads into UID families was performed using UID and mapping position. UID families consisting of less than 3 reads were discarded. Directional adjacency: Clustering of reads was performed using UMI Tools and hence accounting for errors in UIDs. The identified number of ddPCR detected mutations is shown for both clustering methods.

Supplementary Fig. 2 - Comparison of error-rates before and after UID implementation. Left panel: Error-rates calculated per gene panel position. All positions were assigned to bins consisting of 5000 bases and the mean error-rate per bin for all samples is presented. Right panel: Mean error-rates calculated per sample.

Supplementary Fig. 3 - Estimation of detection limit with allocation of more sequencing data. Unique and total reads are displayed for plasma samples amplified using 19 or 20 PCR cycles. Black lines represent individual samples. Blue dots represent mean values for all displayed plasma samples per million total reads. Green dots are based on a model for predicting unique reads from total reads. Representative increments of 10 million total reads are displayed. Dashed lines indicate the overall allocated average of 40 million reads per sample and the hypothetical 100 million reads per sample.

Supplementary table

Supplementary Table 1 – Sequencing and patient details for all plasma samples included in the study.

Supplementary Fig. 1

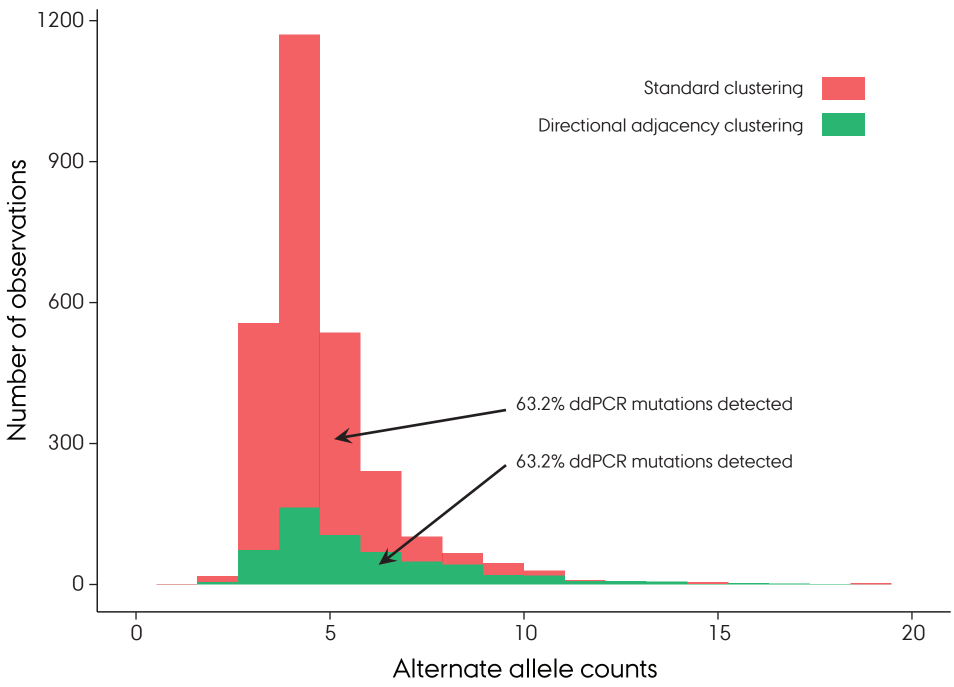

Supplementary Fig. 2

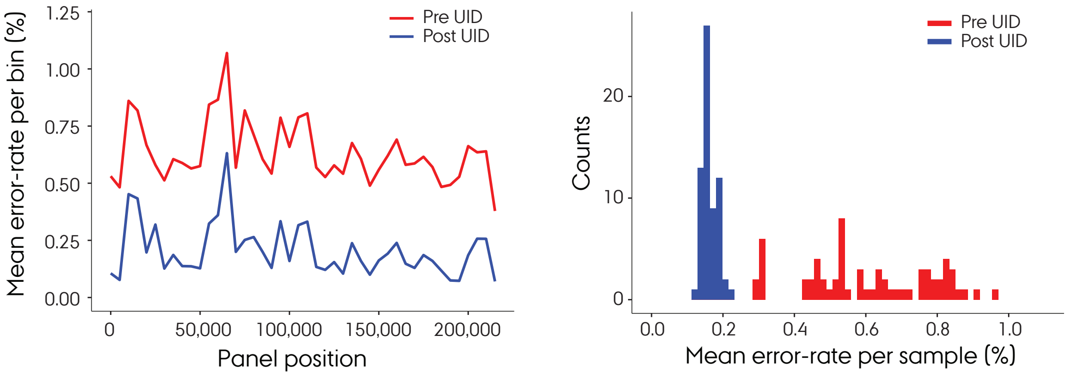

Supplementary Fig. 3

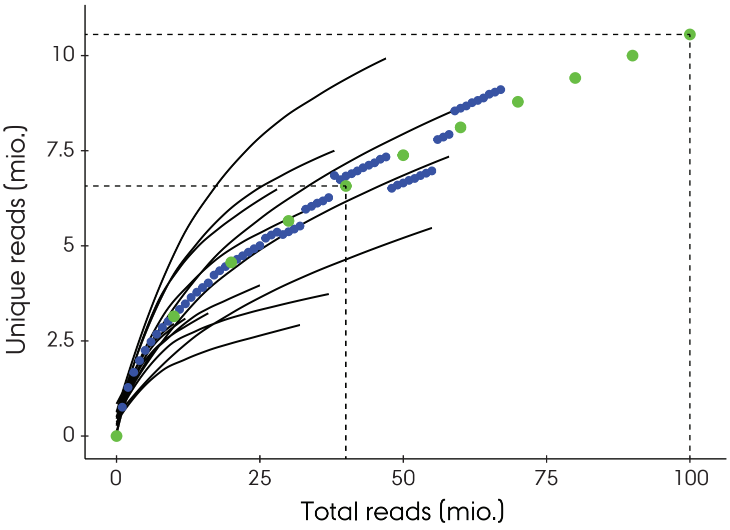

Supplementary Table 1 - Sequencing and patient details for all plasma samples included in the study.

Patient number	Visit number	Plasma volume (mL)	Input DNA (Qubit; ng)	Input DNA (ddPCR; ng)	PCR cycles	PCR split	Plex*	SPRI ratio	Mean UID family size (on-target)	On-target rate (unique reads)	Total reads (mio)	Unique reads (mio)	Unique coverage	Raw coverage
1	6	6,5	25	28	21	1	Multi	1	48,0	0,51	31,2	0,56	89,1	6130
2	12	6,8	52	100	21	1	Single	1	13,4	0,59	26,1	1,49	271,5	5323
2	5	8	42	43	21	1	Single	1	46,9	0,62	65,3	1,26	247,2	17244
3	8	8,6	51	23	25	1	Multi	0,9	53,3	0,59	24,0	0,36	68,4	5358
4	7	7	21	24	21	1	Single	1	39,0	0,48	32,2	0,72	107,9	6095
5	2	8,8	63	46	20	2	Single	1	3,9	0,58	10,5	1,44	259,5	2073
6	10	7	25	42	21	1	Single	1	7,1	0,64	12,4	1,24	244,4	5881
6	12	7	10	47	21	1	Multi	1	38,8	0,61	16,2	0,34	63,0	3554
6	5	7	5	47	21	1	Multi	1	20,1	0,34	9,0	0,28	28,5	965
6	8	7,5	20	29	21	1	Multi	1	38,9	0,63	38,0	0,82	160,6	8682
7	9	13	32	50	21	1	Single	1	31,7	0,56	35,7	0,97	169,2	7375
8	4	8	50	56	21	1	Multi	1	26,4	0,56	42,3	1,34	232,0	8602
8	5	8,6	50	43	25	1	Multi	0,9	53,1	0,56	53,8	0,75	134,9	11276
8	6	4,6	37	23	25	1	Multi	0,9	54,0	0,62	42,1	0,61	121,1	10236
8	7	8,5	25	27	21	1	Multi	1	49,4	0,49	54,4	0,92	141,2	9854
8	8	9	30	47	21	1	Multi	1	49,5	0,52	74,5	1,28	209,0	14800
8	9	8,6	50	44	25	1	Multi	0,9	54,1	0,58	76,2	1,01	183,1	15944
9	6	8,1	50	32	25	1	Multi	0,9	52,8	0,57	21,7	0,32	57,7	4615
9	7	7,2	50	21	25	1	Multi	0,9	56,4	0,57	91,0	1,32	232,4	18233
9	8	8	15	23	21	1	Multi	1	21,7	0,40	21,1	0,67	83,2	2713
9	9	8,6	41	27	25	1	Multi	0,9	52,8	0,60	23,0	0,34	65,5	5221
10	2	7	21	18	21	1	Single	1	24,5	0,60	45,2	1,39	256,8	9973
10	8	13	26	44	21	1	Single	1	20,5	0,62	24,8	0,96	188,7	5929
11	3	8,8	40	29	21	1	Single	1	16,5	0,49	25,0	1,23	190,6	4519
12	2	8,5	79	70	19	2	Single	1	5,0	0,45	27,9	3,31	459,7	4054
13	10	14	44	59	21	1	Single	1	15,9	0,62	29,4	1,38	266,8	6438
13	3	7,5	50	57	19	2	Single	1	6,2	0,56	16,0	1,67	290,6	3196

14	3	8,4	29	20	21	1	Single	1	22,9	0,57	31,6	1,14	202,5	6486
15	13	7	34	58	21	1	Single	1	18,7	0,52	39,8	1,70	277,7	7787
15	3	7,7	50	32	22	1	Multi	1	18,7	0,53	53,0	2,28	375,4	10382
15	8	6,5	56	47	20	2	Single	1	17,6	0,74	32,1	1,49	340,0	9244
16	3	7	26	24	21	1	Single	1	30,8	0,59	29,0	0,81	151,3	6780
17	3	8,7	111	84	21	1	Single	1	8,3	0,60	34,0	2,72	505,8	6800
18	3	8,5	34	29	21	1	Single	1	20,1	0,63	33,2	1,39	276,0	7997
19	10	8	50	61	22	1	Multi	1	19,4	0,50	74,8	3,10	479,2	13644
19	12	8	42	58	21	1	Multi	1	27,0	0,57	52,9	1,62	287,8	11230
19	13	7,5	50	63	21	1	Multi	1	25,6	0,56	39,7	1,24	217,2	8195
19	14	7,5	43	63	21	1	Multi	1	26,7	0,49	40,8	1,16	177,5	6750
19	2	7	25	23	21	1	Multi	1	46,3	0,52	32,7	0,60	98,4	6650
19	5	7	14	21	21	1	Multi	1	22,6	0,39	44,2	1,37	163,4	5424
19	8	7,8	22	34	21	1	Multi	1	22,7	0,43	55,3	1,86	244,3	8025
19	9	7,1	44	20	25	1	Multi	0,9	54,7	0,58	36,6	0,50	92,6	8125
20	10	13	43	66	21	1	Single	1	10,5	0,58	22,0	1,63	292,8	4643
20	2	7,5	36	33	21	1	Single	1	33,0	0,70	48,7	1,23	268,0	13297
21	2	7,7	83	80	20	2	Single	1	13,9	0,72	37,6	2,25	503,1	9985
22	11	11	31	45	21	1	Single	1	17,2	0,58	30,1	1,39	250,7	6419
22	2	6	18	20	21	1	Single	1	46,0	0,71	44,9	0,83	184,1	12674
23	3	8,5	24	24	21	1	Single	1	28,2	0,62	35,2	1,00	194,7	7651
24	3	8,3	36	34	21	1	Single	1	32,8	0,42	64,4	1,55	204,7	11302
25	2	8,5	58	52	20	2	Single	1	18,4	0,43	67,5	2,92	384,7	10566
26	3	9	41	41	21	1	Single	1	27,9	0,49	59,3	1,72	264,8	11446
27	3	9	79	65	21	1	Single	1	14,4	0,47	61,3	3,37	488,0	10573
28	2	10	43	31	20	2	Single	1	23,4	0,52	58,0	1,96	313,0	11343
29	2	8,5	55	52	21	1	Single	1	20,6	0,55	63,3	2,44	427,2	13890
30	2	7,5	24	21	20	2	Single	1	30,2	0,49	54,9	1,36	207,0	10118
31	2	8	53	54	21	1	Single	1	24,5	0,57	66,2	2,23	395,5	14303
32	2	8,5	22	18	21	2	Single	1	14,6	0,29	54,0	2,05	187,3	5086
33	3	4,8	81	85	19	2	Single	1	6,3	0,42	47,5	5,28	672,8	6548
33	3	4,8	85	85	19	4	Single	1	6,7	0,42	38,6	4,16	522,4	5147
34	2	6,9	20	32	21	1	Multi	1	38,7	0,62	63,0	1,33	259,4	13853
34	7	7	41	84	19	2	Single	1	4,7	0,51	12,4	1,52	241,9	2069

35	3	7	43	41	20	2	Single	1	8,2	0,45	34,3	3,10	430,5	5125
36	3	7,5	35	33	20	2	Single	1	9,7	0,51	25,2	2,02	319,6	4472
37	2	8,5	27	26	21	2	Single	1	14,7	0,47	30,1	1,54	222,5	4732
38	2	9	26	30	21	2	Single	1	16,3	0,51	30,5	1,35	212,1	5020

* Plex refers to single sample enrichment or simultaneaous enrichment of multiple samples.
